# Cytoarchitectonic Mapping of MRI Detects Rapid Changes in Alzheimer's Disease

**DOI:** 10.3389/fneur.2020.00241

**Published:** 2020-04-30

**Authors:** Jamie C. Blair, Zofia M. Lasiecka, James Patrie, Matthew J. Barrett, T. Jason Druzgal

**Affiliations:** ^1^Department of Radiology and Medical Imaging, University of Virginia Health System, Charlottesville, VA, United States; ^2^Department of Public Health Sciences, University of Virginia Health System, Charlottesville, VA, United States; ^3^Department of Neurology, University of Virginia Health System, Charlottesville, VA, United States; ^4^Brain Institute, University of Virginia, Charlottesville, VA, United States

**Keywords:** Alzheimer's disease, magnetic resonance imaging, gray matter, atrophy, voxel-based morphometry, atrophy

## Abstract

The clinical and pathological progression of Alzheimer's disease often proceeds rapidly, but little is understood about its structural characteristics over short intervals. This study evaluated the short temporal characteristics of the brain structure in Alzheimer's disease through the application of cytoarchitectonic probabilistic brain mapping to measurements of gray matter density, a technique which may provide advantages over standard volumetric MRI techniques. Gray matter density was calculated using voxel-based morphometry of T1-weighted MRI obtained from Alzheimer's disease patients and healthy controls evaluated at intervals of 0.5, 1.5, 3.5, 6.5, 9.5, 12, 18, and 24 months by the MIRIAD study. The Alzheimer's disease patients had 19.1% less gray matter at 1st MRI, and this declined 81.6% faster than in healthy controls. Atrophy in the hippocampus, amygdala, and basal forebrain distinguished the Alzheimer's disease patients. Notably, the CA2 of the hippocampus was found to have atrophied significantly within 1 month. Gray matter density measurements were reliable, with intraclass correlation coefficients exceeding 0.8. Comparative atrophy in the Alzheimer's disease group agreed with manual tracing MRI studies of Alzheimer's disease while identifying atrophy on a shorter time scale than has previously been reported. Cytoarchitectonic mapping of gray matter density is reliable and sensitive to small-scale neurodegeneration, indicating its use in the future study of Alzheimer's disease.

## Introduction

Understanding alterations to brain structure *in vivo*, which result from neurodegenerative diseases such as Alzheimer's disease (AD), depends on the reliable mapping of the human brain based on the structural distribution of the brain regions (1). To monitor AD *in vivo*, a number of methods to extract regional gray matter features from magnetic resonance images (MRI) through region-of-interest (ROI) analyses exist. These MRI-based methods range from fully automated to “hands-on” manual tracing based on structural landmarks (2). Though automated, the atlas-based methods have improved the interpretation of the results by enabling the standardization of measurements across groups of subjects (3); both automated and manual tracing studies suffer from the frequent use of macroanatomical landmarks which do not correspond directly to cytoarchitectonic borders (2, 4–6).

Early automated ROI analyses were conducted with MRI atlases which were created from a single brain and defined regions based upon gross morphology (7, 8), resulting in susceptibility to inter-individual variability in the physical distribution of the brain regions (7, 9). More recent ROI approaches have addressed brain variability through the ability to assign a probability with which a brain region may be found in stereotaxic space (5, 7, 10, 11). Probabilistic atlases have been created according to region-labeled volumetric MRI, diffusion MRI, functional MRI, MR angiography, or a combination of these modalities (12), but these MRI-based maps often remain susceptible to discordance between macroanatomical landmarks and cytoarchitecture (13). Recent probabilistic maps based on the cytoarchitecture of post-mortem tissue enable the definition of ROIs without a reliance on macroanatomic landmarks (2).

Cytoarchitectonic probabilistic maps are created from non-diseased post-mortem brains which are stained and labeled according to their cell architecture and reconstructed in 3D (10, 14, 15). Each labeled subject map is registered to standard MRI brain space to create a group map representing the relative frequency with which the brain regions are present at individual voxels (7, 16). The microscopic nature of the cytoarchitectonic maps allows for the measurement of regions which are difficult to define macroscropically due to ill-defined boundaries, limited resolution of MRI, or high inter-subject variability (2). Cytoarchitectonic probabilisitic maps have been applied to measurements of brain morphometry in Alzheimer's disease (17), Parkinson's disease (18–20), and healthy aging controls (21–23).

Though calculation of regional volume by manual tracing has been the standard by which AD atrophy is evaluated (24), voxel-based morphometry [VBM; (25)], a technique which automatically calculates gray matter density (GMD) in a voxel-wise manner, has been used extensively to model brain atrophy related to a neurodegenerative disease (26–29). The integration of cytoarchitectonic probabilistic maps and VBM-generated signal intensity allows for the *in vivo* measurement of MRI signal from ROIs which capture interindividual variability in an observer-independent fashion (2). In AD specifically, this technique has been used to demonstrate that basal forebrain degeneration precedes and predicts entorhinal cortex degeneration (17), but usage in AD has been limited relative to studies of Parkinson's disease and normal aging. Due to the promise of this tool for monitoring AD and other neurodegenerative processes, there is a need to understand the reliability and reproducibility of this automated process in both AD patients and controls.

This study applies regional GMD defined by cytoarchitectonic probabilistic maps to AD patients and healthy control (HC) subjects from the Minimal Interval Resonance Imaging in Alzheimer's Disease (MIRIAD) dataset (30). MIRIAD is a publicly available dataset of AD patients and HC subjects which were (1) collected at short intervals and (2) scanned multiple times per session. The reliability and the repeatability of the atrophy measurement techniques have been assessed in this dataset previously, notably in the MIRIAD atrophy challenge, but the probabilistic cytoarchitectonic definition of ROIs was not evaluated (31, 32). Here we hypothesize the reproduction of past structural MRI findings in AD by identifying neurodegeneration in the hippocampal/amygdalar complex (29, 33–35) and basal forebrain (36, 37) and that this method will identify AD-related neurodegeneration on a shorter timescale than has previously been reported. Regional GMD obtained through this method is expected to be reliable and reproducible when obtained over short intervals of time.

## Materials and Methods

### Subjects

#### Healthy Controls

A total of 23 HC subjects were obtained from the MIRIAD database. Briefly, MIRIAD is a single-site longitudinal MRI study of AD conducted at the Dementia Research Center, Institute of Neurology, University College London, UK. MIRIAD was designed to establish the minimal interval with which it would be feasible to conduct clinical trials of AD which used MRI atrophy as an outcome measure (30). HC subjects were included in MIRIAD if they were older than 55 years of age, had a mini-mental state evaluation (MMSE) score of >26/30, and had no history of cognitive impairment, head injury, major psychiatric disease, or stroke. The subjects were excluded from MIRIAD if they had any history of neurodegenerative disease or were unable to tolerate MRI. The HC subjects were scanned at intervals of 0.5, 1.5, 3.5, 6.5, 9.5, 12, 18, and 24 months from baseline. At 0, 1.5, and 9.5 months, two scans were acquired in the same scanning session. Two HC subjects were excluded from analysis in this study due to image quality according to the method discussed below for a final total of 21 subjects included for analysis. Ethical approval for the study was received from the research ethics committee at University College London, and written consent was obtained from all participants.

#### Alzheimer's Disease

A total of 46 patients with a diagnosis of mild-moderate probable AD were obtained from the MIRIAD database. The diagnosis of AD was based on the NINCDS-ADRDA criteria (38). The AD patients were included in MIRIAD if they were over 55 years of age and had a MMSE score between 12 and 26/30. The patients were excluded if they had a history of any neurodegenerative disease besides AD. The AD patients were scanned according to the same longitudinal protocol described for the healthy control subjects. Two AD patients were excluded from the longitudinal analysis due to image quality, for a final total of 44 patients in the longitudinal analysis. Five additional AD patients were excluded from the test–retest analysis in this study due to image quality according to the method discussed below, for a final total of 39 AD patients in the test–retest analysis.

### MRI Acquisition and Quality Control

All MRI images were acquired on the same 1.5-T Signa MRI scanner (GE Medical systems, Milwaukee, WI, USA) by the same MRI radiographer. 3D T1-weighted images were acquired with an inversion recovery prepared fast-spoiled gradient recalled sequence, field of view 24 cm, 256 × 256 matrix, 124 1.5 mm coronal partitions, TR 15 ms, TE 5.4 ms, flip angle 15°, and TI 650 ms. All images analyzed in this study were processed through the automated quality control function contained within the CAT12 toolbox (version r933; http://dbm.neuro.uni-jena.de/cat/) in MATLAB. This tool considers noise, inhomogeneities, and image resolution to create a composite score on a scale of A to E, with A corresponding to the best quality and E corresponding to the worst quality. Only images receiving a composite score of B or higher, corresponding to “good” image quality, were included for a final analysis in this study.

### Image Processing and Region Density Calculation

Regional gray matter density was calculated from AD and HC images according to previously published methods (2). As a note, the use of gray matter “density” or “volume” to describe outputs of voxel-based morphometry is somewhat a source for debate. Here we use the term “gray matter density” to refer to the signal intensity-based output of the method utilized in this study; this choice reflects an attempt to remain consistent with other publications using this specific method. Processing took place in two main steps: (1) preprocessing *via* voxel-based morphometry and (2) application of cytoarchitectonic probabilities to process gray matter density volumes. Voxel-based morphometry (25, 39) was applied to all images from the MIRIAD database using the CAT12 toolbox (http://www.neuro.uni-jena.de/cat/) within SPM12 (Well-come Department of Imaging Neuroscience Group, London, UK; http://www.fil.ion.ucl.ac.uk/spm). The VBM analysis pipeline has been described at length previously (26, 40). Briefly, before processing, the origin of each image was manually reoriented to the anterior commissure in SPM12. Within the CAT12 toolbox, the images were denoised according to spatial-adaptive non-local means denoising (41) and Markov random field (42) approaches. The images were bias-corrected, spatially normalized to standard stereotactic space with an affine registration, and a local intensity transformation was performed. The normalized images were segmented into gray matter, white matter, and cerebrospinal fluid according to the adaptive maximum *a posterior* (AMAP) technique (42). Lorio et al.'s (43) tissue priors were used for spatial normalization, skull stripping, and initial segmentation estimate within the AMAP segmentation. Partial volume estimation (44) estimated partial volume fractions to account for voxels which may contain more than one tissue type. The Diffeomorphic Anatomic Registration Through Exponentiated Lie (45) algorithm as well as Geodesic Shooting (46) was used to register segmented images into standard MNI space. Finally, the segmented images were modulated by the amount of volume changes from the spatial registration to preserve the total amount of gray matter.

**Figure 1:**
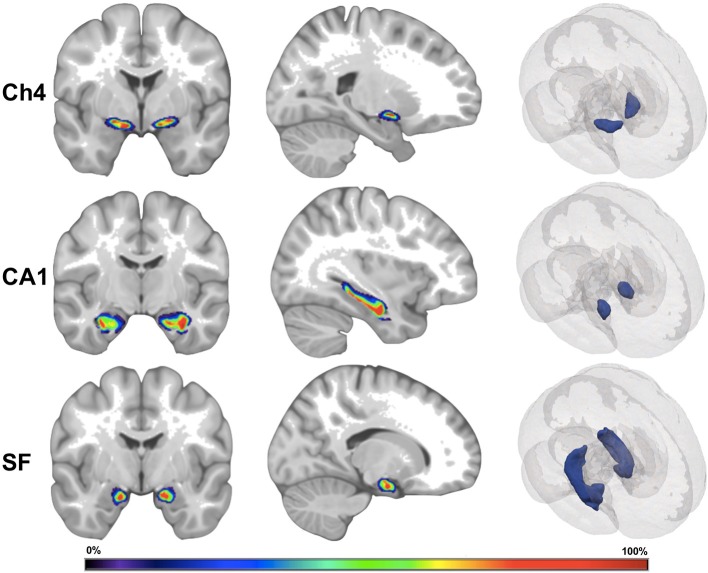
Visual depiction of cytoarchitectonic probabilistic maps. Ch4, nucleus Ch4 of the basal forebrain; CA1, region CA1 of the hippocampus; SF, superficial nucleus of the amygdala. The left and the middle images depict a cytoarchitectonic map superimposed on MNI reference brain. The right images depict cytoarchitectonic maps as 3D mesh inside the MNI reference brain. The color bar corresponds to probability values (0–100%) that a voxel is contained within the cytoarchitectonic map.

Region-specific GMD was measured according to the cytoarchitectonic probabilistic maps for the reference MNI single-subject brain that was derived from the 3D reconstruction of histological sections from post-mortem brains (Figure 1). To apply cytoarchitectonic probabilities to preprocessed gray matter maps, a custom MATLAB script multiplied the value for each voxel in the gray matter map by the weighting contained within the probabilistic map. The weighted GMD values were summed bilaterally and then standardized by dividing each image by the sum of the weighting contained within each probabilistic mask. All cytoarchitectonic probabilistic tissue maps were obtained from the Anatomy Toolbox Version 2.2b and adjusted to correct for normalization into standard MNI space with an affine translation along the y and z axes of 4 and 5 mm (7). The output from this method represents a standardized value for the density of gray matter in a brain region which reflects the likelihood that each voxel in a given brain region will belong to that brain region in a yet untested subject.

In comparing the cytoarchitectonically defined regions available in the Anatomy Toolbox with brain regions implicated in AD by prior MRI studies, 13 subcortical regions and 14 cortical regions were selected. To maintain methodological consistency, only those brain regions for which a cytoarchitectonic brain map had been generated from post-mortem tissue were included for analysis in this study. The available subcortical regions were cholinergic nucleus 4 of the basal forebrain [Ch4; (47)], cholinergic nuclei 1, 2, and 3 of the basal forebrain [Ch1–3; (47)], centromedial amygdala [CM; (15)], laterobasal amygdala [LB; (15)], superficial amygdala [SF; (15)], hippocampal-amygdala transition area [HATA; (15)], amygdala-striatal transition area [ASTR; (15)], entorhinal cortex [EC; (15)], hippocampus area CA1 [CA1; (15)], hippocampus area CA2 [CA2; (15)], hippocampus area CA3 [CA3; (15)], subiculum [SUBC; (15)], and dentate gyrus [DG; (15)]. At the time of analysis, the Anatomy Toolbox contained a list of more than 50 possible neocortical regions to consider. To limit the number of statistical comparisons, we chose a group of 14 neocortical regions: primary motor cortex area 4a [PMC 4a; (48)], primary motor cortex area 4p [PMC 4p; (48)], primary auditory cortex area TE1.0 [TE 1.0; (49)], primary auditory cortex area TE1.1 [TE 1.1; (49)], primary auditory cortex area TE1.2 [TE 1.2; (49)], secondary auditory cortex area TE 3 [TE 3; (50)], primary somatosensory cortex area 1 [PSC 1; (51)], primary somatosensory cortex area 2 [PSC 2; (51)], primary somatosensory cortex area 3a [PSC 3a; (51)], primary somatosensory cortex area 3b [PSC 3b; (51)], Broca's area 44 [BA 44; (52)], Broca's area 45 [BA 45; (52)], occipital cortex area V1 [V1; (53)], and occipital cortex area V2 [V2; (53)]. (Table 1) presents a list of the 27 brain regions measured in this study.

**Table 1:** Complete list of the brain regions included for analysis in this study.

**Brain region**	**Reference**
Ch4 basal forebrain	(47)
Ch1-3 basal forebrain	(47)
Centromedial amygdala	(15)
Laterobasal amygdala	(15)
Superficial amygdala	(15)
Amygdala-striatal transition area	(15)
CA1 hippocampus	(15)
CA2 hippocampus	(15)
CA3 hippocampus	(15)
Dentate gyrus	(15)
Subiculum	(15)
Entorhinal cortex	(15)
Hippocampal-amygdala transition area	(15)
4a primary motor cortex	(48)
4p primary motor cortex	(48)
TE 1.0 primary auditory cortex	(49)
TE 1.1 primary auditory cortex	(49)
TE 1.2 primary auditory cortex	(49)
TE3 secondary auditory cortex	(50)
1 primary somatosensory cortex	(51)
2 primary somatosensory cortex	(51)
3a primary somatosensory cortex	(51)
3b primary somatosensory cortex	(51)
V1 occipital cortex	(53)
V2 occipital cortex	(53)
44 Broca's area	(52)
45 Broca's area	(52)

**Table 2:** Time in months until a.5% decline in gray matter density is detected in each region for the Alzheimer's disease subjects.

**Brain Region**	**All AD Subjects (months)**
Hippo CA2	1.0
Ch4	1.5
Amygdala Astr	1.5
Amygdala SF	1.5
Auditory Te3	1.5
Amygdala CM	2.0
Hippo EC	2.0
Hippo HATA	2.0
Ch123	2.5
Hippo CA1	2.5
Hippo CA3	2.5
Broca BA44	2.5
Broca BA45	2.5
hOC2	2.5
Auditory Te1 2	3.0
Auditory Te1 0	3.5
Amygdala LB	4.0
Auditory Te1 1	4.0
hOC1	4.5
PSC 1	5.0
PSC 3a	6.5
Hippo Subc	7.5
PSC 2	10.0

### Statistical Analyses

Four sets of analyses were conducted which examined the (1) test–retest reliability of cytoarchitectonic probability mapping, (2) differences in baseline GMD between AD patients and HC subjects, (3) rate of change in GMD from 1st MRI between AD patients and HC subjects, and (4) average time from 1st MRI to when regional reductions in GMD of 0.5% can be detected among AD patients.

#### Test–Retest Reliability of Cytoarchitectonic Probabilistic Mapping

Test–retest reliability of cytoachitectonic probabilistic mapping was evaluated by estimating the mean % change in GMD between the 1st and 2nd scans which were conducted within the same scanning session at months 0, 2.5, and 9.5 and by estimating the intraclass correlation (ICC) between all aforementioned within session scans. ICC is a measure of how strongly a set of quantitative measurements acquired from the same entity resemble each other compared to how strongly a set of quantitative measurements acquired from separate entities resemble each other. Values of ICC close to 1 indicate that the measurements acquired from the same entity are highly similar, while values of ICC close to 0 indicated that the measurements acquired from the same entity are highly dissimilar. A global estimate for the average intra-brain-region measurement test–retest reliability of the 1st and the 2nd within-session scans among all brain regions was obtained *via* the image intra-class correlation coefficient I2C2 (54). The I2C2 coefficient is an extension of the aforementioned ICC from the bivariate setting (i.e., test–retest reliability for one brain region) to the multivariate setting (i.e., average test–retest reliability among multiple brain regions). Like the ICC, the I2C2 correlation coefficient has a numeric range: −1 to 1.

A comparison of the mean within-session % change in GMD between the 1st and the 2nd within-session scans between AD patients and HC subjects was conducted by a linear mixed model (LMM) in which the LMM specification was restricted to an intercept parameter that quantitatively estimated the mean % change in GMD between the 1st and the 2nd within-session scans for HC subjects and a parameter that quantitatively estimated the difference between AD patients and HC subjects. One set of hypotheses tested the null hypothesis that the mean % change in GMD between the 1st and the 2nd within-session scans is equal to zero, while the second set of null hypotheses tested that the mean % change in GMD between the 1st and the 2nd within-session scans is the same for AD patients and HC subjects. For both sets of hypothesis tests, the Benjamini and Hochberg false discovery error rate control procedure was used to restrict the false discovery error rate for the entire set of 27 null hypothesis tests to be no >0.05.

With regard to analyzing the degree of homogeneity between the GMD measurements of the 1st and the 2nd within-session scans, the ICC served as the quantitative measure of within-session GMD measurement homogeneity. An ICC was estimated per study group (AD and HC) and brain region combination, and the 95% confidence interval for ICC was derived based on the exact method of Searle. A global estimate for the average intra-brain-region measurement test–retest reliability of the 1st and the 2nd within-session scans among all brain regions was also obtain per study group *via* the I2C2 correlation coefficient, and the 95% confidence interval for the I2C2 was derived by way of the bootstrap resampling procedure of the I2C2 package of R (55).

#### Random Coefficient Regression

Between-group differences in baseline GMD and between-group differences in the rate of change in GMD were examined *via* random coefficient regression (RCR). RCR was selected as the analytical method because the mathematical underpinnings of the RCR model are uniquely suited to modeling correlated repeated-measures-generated response curves as in the current setting (56).

#### Between-Group Differences in Baseline Gray Matter Density

Age- and gender-adjusted comparisons of GMD at baseline were derived by comparing the intercept parameter estimates of a RCR model in which GMD was modeled as a function of time since the 1st MRI (i.e., months), study group (AD and HC), age at 1st MRI, and gender. The RCR model intercept parameter quantitatively estimated the age at 1st MRI and the gender-adjusted mean GMD at the 1st MRI of the HC study population, and the RCR model parameter associated with study group quantitatively estimated the age at 1st MRI and gender-adjusted difference in mean GMD at the 1st MRI between the AD and HC study populations. A simple test of the null hypothesis that the RCR model parameter associated with study group is equal to zero was conducted to compare the mean GMD at the 1st MRI between the AD and the HC study populations. Since this null hypothesis was tested for 27 different brain regions, the Benjamini and Hochberg false discovery error rate procedure was used to restrict the overall false discovery error rate of the entire set of 27 null hypothesis tests to be no >0.05.

#### Between-Group Differences in the Rate of Change in Gray Matter Density

Age- and gender-adjusted comparisons of the rate of change in GMD from the 1st MRI were derived by comparing the slope parameter estimates of the aforementioned random coefficient regression RCR model. The RCR model parameter associated with time since the 1st MRI quantitatively estimated the age at 1st MRI and gender-adjusted mean rate of change in GMD from the 1st MRI for the HC study population, and the RCR model parameter associated with study group by time since the 1st MRI interaction quantitatively estimated the difference between the AD and the HC study populations. A simple test of the null hypothesis that the RCR model parameter associated with study group by time since the 1st MRI interaction is equal to zero was conducted to compare the mean rates of change in GMD from the 1st MRI between the AD and the HC study populations. The Benjamini and Hochberg false discovery error rate procedure was again used to restrict the overall false discovery error rate of the entire set of 27 null hypothesis tests to be no >0.05.

#### Time From the 1st MRI to Detectable Reductions in Gray Matter Density Among Alzheimer's Disease Patients

The average time from the 1st MRI to when regional reductions in GMD of 0.5% can be detected among AD patients was estimated by way of a LMM. The LMM specification included an intercept parameter (β_0_) that quantitatively estimated the mean GMD at the 1st MRI and a slope parameter (β_1_) that quantitatively estimated the expected rate of change in GMD per month of follow-up after the 1st MRI. To estimate the average time required for GMD to be reduced by 0.5% of GMD at the 1st MRI, a 95% confidence interval was constructed for the quantity: 0.005 × β_0_ + β_1_ × [follow-up time (months)]. Using a 0.5 incremental series of follow-up times ranging from 0.5 to 24 months, the average time from the 1st MRI to when a 0.5% reduction in GMD at the 1st MRI is predicted to occur was identified by finding the minimum follow-up time since the 1st MRI such that the 95% confidence interval upper limit for 0.005 × β_0_ + β_1_ × [follow-up time (months)] was <0.

#### Change in Mini-Mental State Evaluation From the 1st MRI

The relationship between MMSE and evaluation time (months) after the 1st MRI was evaluated by RCR, where MMSE served as the dependent variable and the evaluation time after the 1st MRI served as an independent variable along with “study group” (i.e., AD and HC). The AD and HC RCR intercept and slope parameters were compared by way of F-tests, and an F-test was also used to test the null hypothesis that the RCR slope parameter is equal to zero.

#### Change in Mini-Mental State Evaluation as a Function of Change in Gray Matter Density

The relationship between the longitudinal change in MMSE and the longitudinal change in GMD was evaluated by RCR, where the longitudinal change in MMSE served the dependent variable and the longitudinal change in MMSE served as an independent variable along with “study group” (i.e., AD and HC). The null hypothesis that the true underlying slope parameter of the RCR model is equal to zero was tested *via* an F-test separately for each study group. The slope parameters were compared between the AD and the HC patient groups. The Benjamini and Hochberg false discovery error rate procedure was used to restrict the within-study-group and between-study-group overall false discovery error rates of the entire set of 27 null hypothesis tests to be no >0.05.

## Results

GMD measurements from 41 mild–moderate probable AD (24 female, 17 male) and 21 HC (10 female, 11 male) subjects were included for analysis in this study. The age distribution in the AD group was essentially the same [mean = 69.1 years, SD = 6.7 years, range = (55.9, 85.9 years)] as the age distribution in the HC group [mean = 69.3 years, SD = 6.8 years, range = (58.4, 86.0 years)].

### Test–Retest Reliability of Cytoarchitectonic Probabilistic Mapping

**Figure 2:**
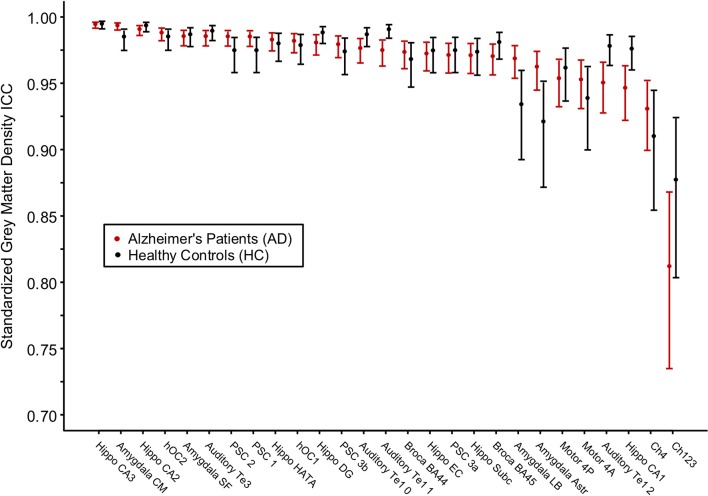
Intraclass correlation coefficients for gray matter density values between two MRI scans within the same scanning session for Alzheimer's disease patients and healthy control subjects. The cytoarchitectonic brain regions are depicted from right to left in order of increasing intraclass correlation value.

For each brain region tested, the mean % change in GMD between all 1st and 2nd within-session scans was calculated for AD patients and HC subjects. Additionally, the difference between mean % change in GMD for within-session scans was compared between AD and HC. No significant differences between same-session scans were detected for AD patients (Table 3) or HC subjects (Table 4). Intra-class correlation coefficients, calculated to examine the similarity between within-session scans, were above >0.8 in each brain region for AD patients and >0.85 in each brain region for HC subjects (Table 5; Figure 2).

**Table 3:** Estimates for the mean % change in the within-session 1st and 2nd standardized gray matter density measurements {i.e., [(2nd – 1^st^)/1^st^]^*^100%} for Alzheimer's disease patients.

**Alzheimer's Disease Patients**						
**Brain region**	**Mean % change**	**Lower 95% CI**	**Upper 95% CI**	***P*****-value**	**B&H threshold**	**Reject**
Auditory Te1 1	−1.15	−1.92	−0.38	0.004	0.00192	No
TE1.0	−1.01	−1.74	−0.27	0.008	0.002	No
TE1.2	−0.95	−1.84	−0.05	0.038	0.00263	No
CA2	−0.81	−1.35	−0.27	0.004	0.00185	No
BA45	−0.76	−1.36	−0.16	0.014	0.00208	No
SUBC	−0.68	−1.33	−0.03	0.042	0.00278	No
DG	−0.67	−1.29	−0.05	0.035	0.00238	No
TE3.0	−0.62	−1.2	−0.05	0.035	0.00227	No
V1	−0.56	−1.19	0.06	0.077	0.00294	No
BA44	−0.56	−1.28	0.16	0.124	0.00333	No
LB	−0.55	−1.7	0.6	0.339	0.00455	No
V1	−0.52	−1.01	−0.03	0.037	0.0025	No
CA1	−0.51	−1.48	0.46	0.298	0.00417	No
PSC 3a	−0.48	−1.28	0.33	0.243	0.00385	No
CA3	−0.46	−0.85	−0.07	0.02	0.00217	No
EC	−0.34	−1.33	0.64	0.486	0.00556	No
HATA	−0.29	−0.9	0.33	0.355	0.005	No
SF	−0.13	−1.27	1.01	0.82	0.01	No
PSC 3b	−0.02	−0.75	0.7	0.947	0.01667	No
PSC 2	0.01	−0.81	0.83	0.987	0.05	No
CM	0.01	−0.5	0.52	0.962	0.025	No
PSC 1	0.03	−0.67	0.73	0.93	0.0125	No
Motor 4P	0.2	−0.57	0.97	0.607	0.00625	No
Ch4	0.27	−1.06	1.6	0.688	0.00833	No
ASTR	0.33	−1.22	1.88	0.673	0.00714	No
Motor 4A	0.67	−0.1	1.44	0.087	0.00313	No
Ch123	1.24	−0.35	2.83	0.125	0.00357	No

**Table 4:** Estimates for the mean % change in the within-session 1st and 2nd standardized gray matter density measurements {i.e. [(2nd – 1^st^)/1^st^]^*^100%} for the healthy control subjects.

**Healthy Controls**						
**Brain region**	**Mean % change**	**Lower 95% CI**	**Upper 95% CI**	***P*****-value**	**B&H threshold**	**Reject**
TE1.2	−1.15	−1.97	−0.33	0.007	0.00192	No
TE1.0	−0.76	−1.36	−0.15	0.016	0.00208	No
TE1. 1	−0.53	−1.14	0.07	0.084	0.00278	No
TE3.0	−0.45	−0.78	−0.12	0.009	0.002	No
Ch123	−0.44	−1.86	0.99	0.538	0.00556	No
BA45	−0.36	−0.96	0.24	0.228	0.00357	No
BA44	−0.35	−1	0.29	0.276	0.00385	No
SF	−0.33	−0.71	0.05	0.083	0.00263	No
HATA	−0.21	−0.85	0.44	0.519	0.005	No
SUBC	−0.14	−0.63	0.36	0.579	0.00625	No
V1	−0.13	−0.65	0.39	0.616	0.01	No
V2	−0.11	−0.53	0.31	0.603	0.00833	No
EC	−0.1	−0.73	0.52	0.739	0.01667	No
CA3	−0.06	−0.34	0.21	0.636	0.0125	No
CA2	−0.05	−0.4	0.29	0.748	0.025	No
LB	0.05	−0.81	0.91	0.906	0.05	No
PSC 3a	0.17	−0.46	0.8	0.596	0.00714	No
DG	0.2	−0.13	0.53	0.227	0.00333	No
CM	0.31	−0.1	0.71	0.133	0.00294	No
PSC 1	0.38	−0.4	1.17	0.332	0.00417	No
ASTR	0.4	−0.64	1.45	0.438	0.00455	No
PSC 2	0.43	−0.2	1.05	0.176	0.00313	No
CA1	0.54	0.04	1.03	0.034	0.00227	No
PSC 3b	0.67	−0.02	1.36	0.057	0.00238	No
Motor 4P	0.95	−0.08	1.98	0.071	0.0025	No
Motor 4A	1.05	0.35	1.75	0.004	0.00185	No
Ch4	1.35	0.17	2.53	0.026	0.00217	No

**Table 5:** Intra-class correlation coefficients examining the similarity between replicate standardized gray matter density measurements for the Alzheimer's disease patients and for the healthy control subjects.

	**Alzheimer's disease patients**			**Healthy controls**		
**Brain region**	**ICC**	**Lower 95% CI**	**Upper 95% CI**	**ICC**	**Lower 95% CI**	**Upper 95% CI**
Ch4	0.93	0.9	0.95	0.91	0.85	0.94
Ch123	0.81	0.73	0.87	0.88	0.8	0.92
ASTR	0.96	0.94	0.97	0.92	0.87	0.95
CM	0.99	0.99	1	0.98	0.97	0.99
LB	0.97	0.95	0.98	0.93	0.89	0.96
SF	0.99	0.98	0.99	0.99	0.98	0.99
CA1	0.95	0.92	0.96	0.98	0.96	0.99
CA2	0.99	0.99	0.99	0.99	0.99	1
CA3	0.99	0.99	1	0.99	0.99	1
DG	0.98	0.97	0.99	0.99	0.98	0.99
EC	0.97	0.96	0.98	0.97	0.96	0.98
HATA	0.98	0.97	0.99	0.98	0.97	0.99
SUBC	0.97	0.96	0.98	0.97	0.96	0.98
TE3.0	0.99	0.98	0.99	0.99	0.98	0.99
TE1.0	0.98	0.97	0.98	0.99	0.98	0.99
TE1.1	0.97	0.96	0.98	0.99	0.98	0.99
TE1.2	0.95	0.93	0.97	0.98	0.96	0.99
BA44	0.97	0.96	0.98	0.97	0.95	0.98
BA45	0.97	0.96	0.98	0.98	0.97	0.99
Motor 4A	0.95	0.93	0.97	0.94	0.9	0.96
Motor 4P	0.95	0.93	0.97	0.96	0.94	0.98
PSC 1	0.98	0.98	0.99	0.97	0.96	0.98
PSC 2	0.99	0.98	0.99	0.97	0.96	0.98
PSC 3a	0.97	0.96	0.98	0.97	0.96	0.98
PSC 3b	0.98	0.97	0.99	0.97	0.96	0.98
V1	0.98	0.97	0.99	0.98	0.96	0.99
V2	0.99	0.98	0.99	0.98	0.97	0.99

A global estimate for the average intra-brain-region measurement test–retest reliability of the 1st and the 2nd within-session scans among all brain regions was obtained *via* the image intra-class correlation coefficient I2C2: AD [0.976, 95%CI (0.969, 0.982)], HC [0.973, 95%CI (0.967, 0.978)], and combined AD and HC [0.988, 95%CI (0.986, 0.990)]. The IC2C estimate values all exceeded 0.95.

To further test the measurement agreement of the 1st and the 2nd within-scan session, a Bland–Altman analysis was conducted with the output expressed as a ratio of the 2nd scan measurement to the 1st scan measurement of GMD (Supplementary Figure 1). The geometric mean ratios for AD patients fell between 0.986 and 1.012 units and did not differ significantly from the null hypothesis. The geometric mean ratios for HC subjects fell between 0.985 and 1.015 units and did not differ significantly from the null hypothesis.

### Between-Group Baseline Differences in Gray Matter Density

**Figure 3:**
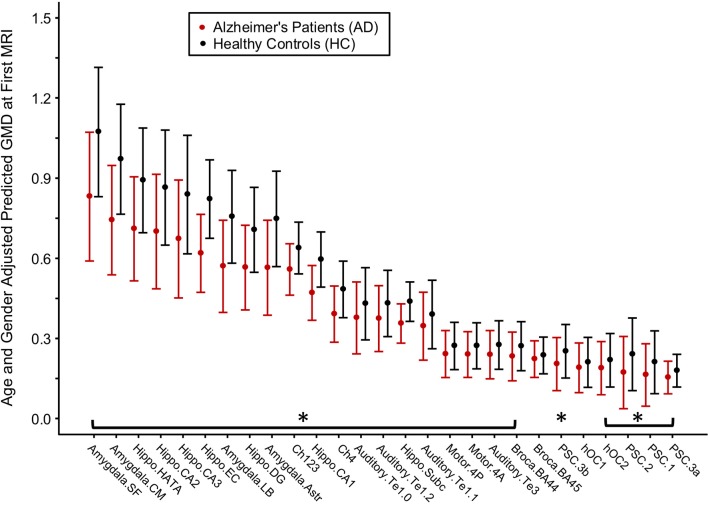
Differences in gray matter density (GMD) between Alzheimer's disease (AD) patients and healthy control (HC) subjects at baseline. Age- and gender-adjusted GMD values at first MRI are depicted from left to right in order of increasing decline in GMD per month for the AD subjects. Asterisks denote the age- and gender-adjusted GMD at first MRI that is significantly different between AD and HC at *p* < 0.05 corrected by Benjamini and Hochberg false discovery rate correction.

For each brain region tested, age- and gender-adjusted mean GMD was compared between AD and HC groups at baseline (Figure 3). The age- and gender-adjusted mean GMD differed between AD and HC for 25 of the 27 regions; only Broca's area 45 and occipital cortex area V1 were not different between the groups. The three regions with the largest difference between AD and HC were the centromedial amygdala (23.5% less GMD for AD than HC), superficial amygdala (22.6% less GMD for AD than HC), and the hippocampal-amygdala transition area (20.4% less GMD for AD than HC).

### Between-Group Differences in Gray Matter Density Rate of Change

**Figure 4:**
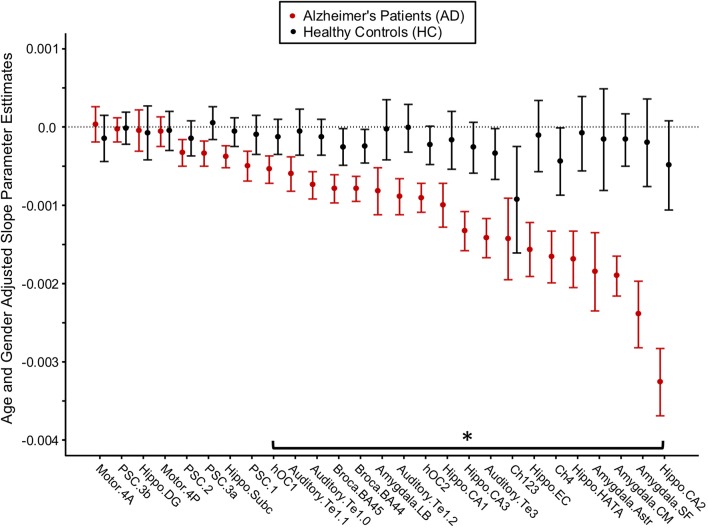
Longitudinal change in gray matter density (GMD) in Alzheimer's disease (AD) patients and healthy control (HC) subjects. Age- and gender-adjusted slope parameter estimates for the change in GMD per month are depicted from left to right in order of worsening decline in GMD per month for the AD subjects. The asterisk denotes the change in GMD per month that is significantly different between AD and HC at *p* < 0.05, FDR corrected.

For each brain region, age- and gender-adjusted cohort comparisons of the predicted slope for the monthly change in GMD from the 1st MRI were calculated (Figure 4). The age- and gender-adjusted slopes were significantly different between AD and HC for 19 of the 27 selected regions. The three regions with the largest difference between AD and HC were hippocampus area CA2 (85% faster for AD than HC), centromedial amygdala (declined 91.6% faster for AD than HC), and superficial amygdala (declined 91.6% faster for AD than HC).

### Minimum Interval to Detect Regional Change

For AD patients, the estimated time required to detect a 0.5% reduction in GMD from the 1st MRI (baseline) was calculated (Table 2). A reduction in GMD >0.5% from baseline was detected in 23 of the 27 tested regions. Only the dentate gyrus, primary somatosensory cortex area 3b, and primary motor cortex areas 4a and 4p did not lose 0.5% of baseline GMD over the first 24 months of follow-up. The regions for which GMD declined by 0.5% from baseline during the study period were area CA2 of the hippocampus (1.0 month), cholinergic nucleus 4 (1.5 months), amygdala-striatal transition area (1.5 months), superficial amygdala (1.5 months), secondary auditory cortex area TE3 (1.5 months), centromedial amygdala (2.0 months), entorhinal cortex (2.0 months), hippocampal-amygdala transition area (2.0 months), cholinergic nucleus 1–3 (2.5 months), area CA1 of the hippocampus (2.5 months), area CA3 of the hippocampus (2.5 months), Broca's area 44 (2.5 months), Broca's area 45 (2.5 months), occipital cortex area V2 (2.5 months), area TE 1.2 of the auditory cortex (3.0 months), area TE 1.0 of the auditory cortex (3.5 months), laterobasal amygdala (4.0 months), area TE 1.1 of the auditory cortex (4.0 months), occipital cortex area V1 (4.5 months), area 1 of the primary somatosensory cortex (5.0 months), area 3a of the primary somatosensory cortex (6.5 months), subiculum (7.5 months), and area 2 of the primary somatosensory cortex (10.0 months).

#### Change in Mini-Mental State Evaluation From the 1st MRI

The relationship between MMSE and months after the 1st MRI was evaluated by random coefficient regression. Months after the 1st MRI predicted MMSE in the Alzheimer's disease patients [−0.23, 95%CI (−0.29, −0.17), *p* < .001] but not in the healthy control subjects [0.00, 95%CI (−0.08, 0.8), *p* = 1.00] (Supplementary Table 2).

#### Change in Mini-Mental State Evaluation as a Function of Change in Gray Matter Density

The relationship between the longitudinal change in MMSE and the longitudinal change in GMD was evaluated by random coefficient regression. In Alzheimer's disease patients, MMSE was associated with the longitudinal change in GMD in 15 of the 27 brain regions tested (Supplementary Table 1, Supplementary Figure 2). In healthy control subjects, MMSE was not associated with longitudinal change in GMD in any brain region tested (Supplementary Table 1). The between-group differences between AD patients and HC subjects were non-significant (Supplementary Table 1).

## Discussion

The cytoarchitectonic probabilistic mapping of GMD allows for the evaluation of brain structures affected by a neurodegenerative disease. For both AD patients and HC subjects, the regional GMD measurements obtained with this method are stable—in both the cortical and the subcortical structures—across repeated measurements in a single MRI scanning session. This result indicates that the method produces very reproducible measurements in an idealized MRI scanning scenario. In baseline data from the same cohorts, widespread regional GMD differences separated the AD and the HC groups, particularly in the subcortical structures (*e.g*., hippocampus, amygdala, and cholinergic nuclei of the basal forebrain). A longitudinal analysis over the 2-year study period supports a complementary narrative. In the HC group, the regional GMD measurements remained relatively stable over 2 years, allowing that some degree of neurodegeneration happens during “healthy” aging. In contrast, the AD patients showed a widespread longitudinal decline in regional GMD with a preferentially subcortical pattern resembling the group differences at baseline. Multiple brain regions showed measurable changes in 3 months or less. These findings aggregately suggest that the cytoarchitectonic mapping of GMD can measure short-interval changes in regional GMD that are not occurring in healthy aging controls.

A number of studies have investigated the reliability of tools that measure brain volume and morphometry such as Freesurfer and FSL (31, 32, 57–59). To date, the reliability of cytoarchitectonic probabilistic mapping of GMD has not been evaluated. The ICC values reported here, all >0.9 with the exception of the cholinergic nuclei of the basal forebrain—which exceed.8—are in agreement with past studies of Freesurfer's cortical and subcortical parcellations (57, 59). The Freesurfer automated pipelines, which have been well validated and are widely accepted for use in the study of neurodegenerative diseases (60), represent a benchmark by which to measure reliability. Prior studies of Freesurfer's subcortical parcellation identified the hippocampal-amygdala transition area as a region of lower reliability, which is supported by work identifying the HATA as an area of high inter-subject variability (15, 57). Here we report ICC values >0.95 for HATA in both AD patients and HC subjects, indicating the resilience of this tool for high-variability brain regions. Though Figure 2 appears to demonstrate a difference in variability between HC subjects and AD patients such that HC measurements are less reliable, this is likely due to the limited number of HCs rather than a feature of this technique.

Morphometric MRI studies of AD are extensive in the literature, with results demonstrating a consistent pattern of atrophy localized to the hippocampus (61, 62), amygdala (63, 64), basal forebrain (37, 65), thalamus (66), and neocortex (67, 68). Despite differences in the analysis technique, findings from this study largely support prior morphometric and volumetric studies of AD. In the hippocampal formation specifically, density was 20.6% lower in AD than in HC (95%CI: 17.0, 26.1%), closely mirroring manual tracing studies that place the difference between mild AD and HC between 15 and 30% (35). The entorhinal cortex showed the largest difference between the groups at baseline, a finding supported by its status as a site of early infiltration for tau pathology in the Braak model (69).

This study reports that the rates of decline as measured by the change in GMD per month agree with the baseline cross-sectional findings—that is, regions which are most different between AD patients and HC subjects at baseline also decline more quickly over time. The hippocampus, amygdala, and basal forebrain declined more quickly in AD patients than in HC subjects, and the subcortical regions declined more quickly than the neocortical brain regions. These findings are supported by the Braak model, which suggests that mature neocortical neurons are not affected by pathology until later in the disease process (69). Interestingly, we report that the amygdala declined 11.6% faster than the hippocampus (95%CI 9.9, 13.0%) in AD patients in this study. The amygdala volume has been strongly implicated in AD (70), but studies concerning the rate of decline relative to the hippocampus have been inconsistent, with the amygdala showing lower levels of atrophy (71, 72), equal levels of atrophy (73), and greater levels of atrophy as shown in this study (74). A recent multisite lifespan study of AD identified the amygdala as an area of divergence from controls as early as age 40, highlighting its key role in the development of AD (75). Taken together, these findings suggest the amygdala as an area of future study in AD.

A novel finding of this study is the short interval at which the cytoarchitectonic mapping of GMD was able to detect AD-related change. In the CA2 of the hippocampus, we detected a.5% decrease in GMD in only 1 month post-baseline, a finding which disagrees with a prior longitudinal study of the MIRIAD dataset (57). A previous work failed to detect any significant changes in hippocampal volume over a 6-week period, and though it did identify significant atrophy to the whole hippocampus over a 2-year period, it did not identify atrophy specific to the CA2 (57). This discrepancy is likely related to the difference in analysis technique; Worker et al. extracted hippocampal volumes from the Freesurfer hippocampal subfield pipeline, which uses an atlas based on high-resolution MRI (76) rather than cytoarchitectonic maps. The key involvement of the CA2 identified in this study is supported by Braak, which implicates the CA2 early in the staging model of AD alongside the CA1 and ahead of the other subregions of the hippocampus (69). Future work will explore the potential of the CA2 as a longitudinal marker of AD.

One remaining question from these results is why the orderly progression of AD in the hippocampus was not observed. Braak and Braak (77) previously noted an organized progression of neurofibrillary tangles beginning in the CA1 and later emerging in the subiculum, CA2, CA3, and CA4. Additionally, decreased functional connectivity in the CA1 and the CA2 has been noted in patients with mild cognitive impairment (78). Here we noted the degeneration of the CA2 and the subregions of the amygdala at much faster rates than the CA1 and subiculum. We believe that this finding is due to the heterogeneous nature of the subjects collected as part of the MIRIAD study. Subjects were included in MIRIAD if they were over the age of 55 years, had a diagnosis of mild–moderate probable AD, and had a MMSE between 12 and 26/30 (30). These criteria likely led to the inclusion of some “early” and some “more advanced” AD patients, making visualization of orderly progression difficult. The method outlined in this study is capable of measuring rapid change in GMD, and a different cohort of MCI or “early” AD patients may return findings which more closely adhere to those hippocampal subfields noted by Braak and Braak (77).

Though considered as strength of the MIRIAD dataset, the homogenous collection of images on the same 1.5-T scanner by the same MRI technologist limited our ability to make a wider inference about the reliability of this technique in other longitudinal MRI datasets. It is known that factors such as field strength (79), acquisition parameters (80), and individual MRI scanner (81) all influence the accuracy and the reliability of VBM. Though large neuroimaging databases such as the Alzheimer's Disease Neuroimaging Initiative (ADNI) and the Parkinson's Progression Markers Initiative seek to standardize their imaging protocols, the same participant may be scanned in multiple MRI scanners by different radiographers and sometimes with variable acquisition parameters over time. It is probable that the reliability of this technique would be lower for less homogenous longitudinal studies due to the increased variability in factors that influence the VBM outcomes. Ideally, we would have integrated additional datasets, such as ADNI, to demonstrate this point. However, the significantly longer interval at which ADNI and other longitudinal MRI datasets collect data precluded this possibility. Through a multi-site study, future work could explore the influence of scanner and technologist variability on the short-interval reliability of this technique.

## Conclusion

The current study provides evidence for the cytoarchitectonic mapping of gray matter density as an important tool for the analysis of neurodegenerative diseases. Average intraclass correlation coefficients >0.9 for both AD patients and HC subjects coupled with sensitivity for AD-related atrophy on a shorter timescale than has previously been reported underscore the power of histologically defined brain maps for the *in vivo* evaluation of AD and other diseases. Ongoing research efforts will apply this technique to additional disease processes for the detection of sub-regional atrophy.

## Data Availability Statement

All data analyzed in this study were obtained from the publicly available MIRIAD dataset. The data can be accessed at https://www.ucl.ac.uk/drc/research/methods/minimal-interval-resonance-imaging-alzheimers-disease-miriad.

## Ethics Statement

The studies involving human participants were reviewed and approved by the the research ethics committee at University College London, and written consent was obtained from all participants. The patients/participants provided their written informed consent to participate in this study.

## Author Contributions

JB, ZL, MB, and TD contributed to the study concept and design. JB and ZL contributed to the acquisition of data. JP conducted the statistical analysis. JB, ZL, JP, and TD contributed to the analysis and interpretation of data. JB and TD drafted the manuscript. ZL, JP, and MB contributed to the critical revision of the manuscript for intellectual content.

## Conflict of Interest

The authors declare that the research was conducted in the absence of any commercial or financial relationships that could be construed as a potential conflict of interest.
