# Supplementary material for: Cytoarchitectonic Mapping of MRI Detects Rapid Changes in Alzheimer's Disease
Source: Front Neurol. 2020 Apr 30;11:241. doi: 10.3389/fneur.2020.00241 (PMC7203491; doi:10.3389/fneur.2020.00241)
Supplement: Supplementary file 1 [file Data_Sheet_1.docx]

|  |  | AD Patients | | | |  |  |  |  | Healthy |  |  | AD vs HC | | |
| --- | --- | --- | --- | --- | --- | --- | --- | --- | --- | --- | --- | --- | --- | --- | --- |
| Brain Region | Slope | [ 95% CI ] | | P-value | B&H  Threshold | Reject | **Slope** | [ 95% CI ] | | P-value | B&H  Threshold | Reject | P-value | B&H  Threshold | Reject |
| Broca BA45 | 154.794 | 82.785 | 226.803 | 0.00017 | 0.00238 | Yes | -19.038 | -151.026 | 112.951 | 0.77317 | 0.00294 | No | 0.77317 | 0.00294 | No |
| Broca BA44 | 133.160 | 65.540 | 200.781 | 0.00055 | 0.00278 | Yes | -3.605 | -119.367 | 112.156 | 0.95017 | 0.00833 | No | 0.95017 | 0.00833 | No |
| Auditory Te3 | 130.958 | 79.050 | 182.866 | 0.00002 | 0.00208 | Yes | -22.474 | -115.995 | 71.048 | 0.62971 | 0.00227 | No | 0.62971 | 0.00227 | No |
| Amygdala CM | 111.139 | 74.387 | 147.891 | 0.00000 | 0.00185 | Yes | -1.432 | -71.431 | 68.567 | 0.96766 | 0.01000 | No | 0.96766 | 0.01000 | No |
| Auditory Te1 1 | 101.285 | 53.092 | 149.479 | 0.00021 | 0.00250 | Yes | -8.250 | -93.609 | 77.109 | 0.84747 | 0.00455 | No | 0.84747 | 0.00455 | No |
| Hippo CA3 | 97.874 | 46.288 | 149.461 | 0.00066 | 0.00294 | Yes | 18.230 | -88.962 | 125.422 | 0.73688 | 0.00263 | No | 0.73688 | 0.00263 | No |
| PSC 2 | 92.295 | 12.738 | 171.853 | 0.02452 | 0.00556 | No | -1.136 | -99.778 | 97.506 | 0.98130 | 0.01250 | No | 0.98130 | 0.01250 | No |
| Auditory Te1 0 | 80.061 | 33.329 | 126.793 | 0.00136 | 0.00357 | Yes | 8.746 | -81.858 | 99.349 | 0.84857 | 0.00500 | No | 0.84857 | 0.00500 | No |
| Ch4 | 77.860 | 47.441 | 108.279 | 0.00007 | 0.00217 | Yes | -0.177 | -38.636 | 38.282 | 0.99254 | 0.02500 | No | 0.99254 | 0.02500 | No |
| PSC 1 | 77.837 | 25.750 | 129.923 | 0.00379 | 0.00385 | Yes | 6.335 | -56.427 | 69.097 | 0.84011 | 0.00417 | No | 0.84011 | 0.00417 | No |
| hOC2 | 77.754 | 20.799 | 134.708 | 0.00986 | 0.00500 | No | -11.852 | -115.715 | 92.011 | 0.82022 | 0.00385 | No | 0.82022 | 0.00385 | No |
| Auditory Te1 2 | 77.139 | 33.948 | 120.329 | 0.00095 | 0.00313 | Yes | 5.299 | -84.297 | 94.894 | 0.90659 | 0.00556 | No | 0.90659 | 0.00556 | No |
| Hippo EC | 75.271 | 45.881 | 104.660 | 0.00001 | 0.00200 | Yes | -1.392 | -42.034 | 39.250 | 0.94461 | 0.00714 | No | 0.94461 | 0.00714 | No |
| PSC 3a | 70.034 | -15.371 | 155.439 | 0.10336 | 0.01250 | No | 48.390 | -87.505 | 184.284 | 0.47527 | 0.00185 | No | 0.47527 | 0.00185 | No |
| Hippo Subc | 68.171 | -6.229 | 142.571 | 0.07108 | 0.00833 | No | 14.050 | -102.652 | 130.752 | 0.81140 | 0.00357 | No | 0.81140 | 0.00357 | No |
| Hippo CA2 | 65.247 | 41.931 | 88.562 | 0.00000 | 0.00192 | Yes | 0.129 | -56.330 | 56.588 | 0.99640 | 0.05000 | No | 0.99640 | 0.05000 | No |
| Amygdala SF | 49.880 | 27.132 | 72.627 | 0.00016 | 0.00227 | Yes | -15.111 | -66.429 | 36.207 | 0.54864 | 0.00208 | No | 0.54864 | 0.00208 | No |
| Hippo HATA | 49.072 | 15.662 | 82.483 | 0.00568 | 0.00417 | No | -11.612 | -58.929 | 35.704 | 0.61929 | 0.00217 | No | 0.61929 | 0.00217 | No |
| Amygdala Astr | 37.282 | 16.306 | 58.258 | 0.00128 | 0.00333 | Yes | 4.133 | -28.440 | 36.707 | 0.79484 | 0.00313 | No | 0.79484 | 0.00313 | No |
| Hippo CA1 | 33.451 | -21.849 | 88.751 | 0.22625 | 0.01667 | No | 0.676 | -95.870 | 97.222 | 0.98888 | 0.01667 | No | 0.98888 | 0.01667 | No |
| Ch123 | 32.342 | 14.528 | 50.157 | 0.00046 | 0.00263 | Yes | -5.010 | -36.963 | 26.944 | 0.75729 | 0.00278 | No | 0.75729 | 0.00278 | No |
| Amygdala LB | 21.075 | -4.331 | 46.482 | 0.09889 | 0.01000 | No | -8.593 | -50.884 | 33.698 | 0.67239 | 0.00238 | No | 0.67239 | 0.00238 | No |
| hOC1 | 10.385 | -45.433 | 66.203 | 0.69863 | 0.05000 | No | -14.080 | -129.792 | 101.632 | 0.81001 | 0.00333 | No | 0.81001 | 0.00333 | No |
| PSC 3b | -33.532 | -142.415 | 75.352 | 0.53599 | 0.02500 | No | 7.239 | -136.347 | 150.825 | 0.91885 | 0.00625 | No | 0.91885 | 0.00625 | No |
| Motor 4P | -36.540 | -73.454 | 0.374 | 0.05229 | 0.00714 | No | 25.046 | -56.769 | 106.860 | 0.54541 | 0.00200 | No | 0.54541 | 0.00200 | No |
| Hippo DG | -39.866 | -76.545 | -3.188 | 0.03364 | 0.00625 | No | 15.516 | -62.259 | 93.291 | 0.69338 | 0.00250 | No | 0.69338 | 0.00250 | No |
| Motor 4A | -66.867 | -113.169 | -20.566 | 0.00956 | 0.00455 | No | 18.739 | -42.151 | 79.630 | 0.54006 | 0.00192 | No | 0.54006 | 0.00192 | No |

Supplementary Table 1. Random coefficient regression slope parameter estimates for predicting the longitudinal change in MMSE (Δ MMSE) as a function of the longitudinal change in the GMD score (Δ GMD) and subject age, where subject age was set at 70 years of age.

| Regression Parameter | Parameter  Estimate | Lower 95%  CL | Upper 95%  CL | P-value |
| --- | --- | --- | --- | --- |
| Intercept AD | 20.174 | 19.093 | 21.255 |  |
| Slope AD | -0.229 | -0.290 | -0.169 | 0.000 |
| Intercept Healthy Control | 29.368 | 27.895 | 30.841 |  |
| Slope Heathy Control | 0.000 | -0.080 | 0.080 | 1.000 |
| Comparison | Mean  Difference | Lower 95%  CL | Upper 95%  CL | P-value |
| Intercept (Healthy Control – AD) | 9.194 | 7.367 | 11.021 | <0.001 |
| Slope (Healthy Control – A) | 0.229 | 0.129 | 0.330 | <0.001 |

Supplementary Table 2. Random coefficient regression parameter estimates for predicting MMSE as a function of the number of months since 1^st^ MRI exam.


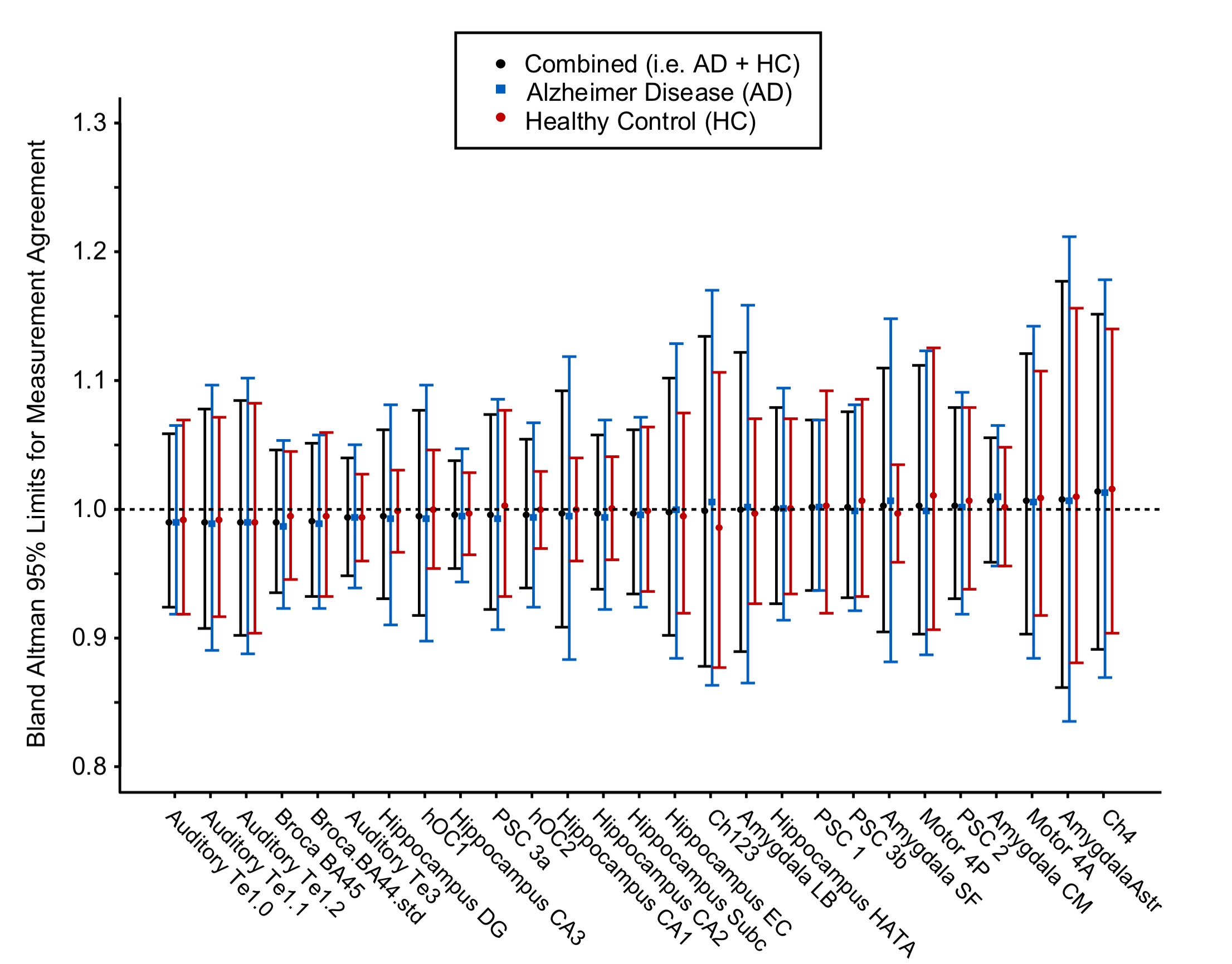


Supplementary Figure 1: Bland-Altman measurement agreement summaries expressed as a ratio of the 2^nd^ scan measurement to 1^st^ scan measurement of GMD. Dots identify the ratio of geometric GMD means (i.e. 2^nd^ scan: 1^st^ scan). Vertical lines identify the range of values between the lower 95% confidence limit for the Bland-Altman lower limit of agreement, and the upper 95% confidence limit for the Bland-Altman upper limit of agreement.


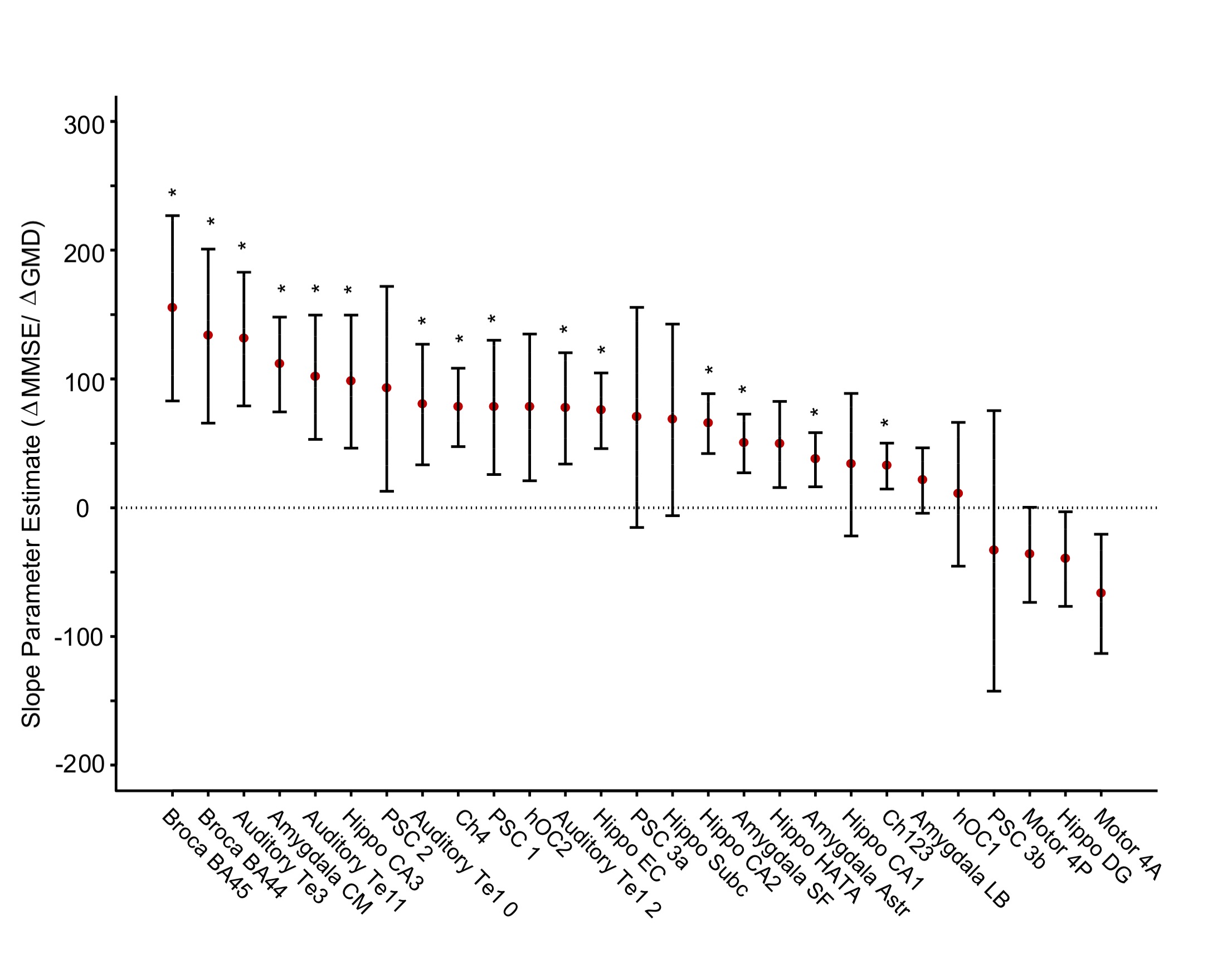


Supplementary Figure 2: Random coefficient regression slope parameter estimates for predicting the change in MMSE (Δ MMSE) as a linear function of the corresponding change in the GMD (Δ GMD) in AD patients. Vertical lines identify the 95% confidence interval for the slope parameter and asterisks identify rejections of the null hypothesis.
